# Information quality of videos related to adolescent depression on social media platforms: a comparative study of TikTok and BiliBili

**DOI:** 10.3389/fpubh.2025.1663977

**Published:** 2025-11-19

**Authors:** Guiyun Wang, Jinke Kuang, Yanxia Qi, Jingya Li

**Affiliations:** 1 Shandong Xiehe University, Jinan, Shandong, China; 2 Shandong Mental Health Center, Jinan, Shandong, China

**Keywords:** adolescents depression, social media, quality analysis, TikTok, Bilibili

## Abstract

**Background:**

The incidence of adolescent depression has been increasing globally in recent years, raising concern among the public about this condition. Videos on adolescent depression are disseminated through TikTok and Bilibili, both of which have gained popularity in recent years as easily accessible sources of health information. However, no researchers have conducted professional inspection and evaluation of depression-related videos targeting adolescents, some of these videos may even disseminate misleading information.

**Methods:**

We retrieved the top 100 adolescent depression related videos from TikTok and Bilibili. Data on video characteristics, including engagement metrics and content, were also collected. Video quality was assessed using three rating tools: the Journal of the American Medical Association (JAMA), Global Quality Score (GQS), and the Modified DISCERN (mDISCERN). The independent *t*-test, Mann–Whitney *U* test, and Kruskal–Wallis test were used for comparison and analysis.

**Results:**

The analysis included 188 videos, with 95 from TikTok and 93 from Bilibili. TikTok videos were shorter and exhibited higher audience interaction. The most popular topic on TikTok and Bilibili was “Symptoms of adolescent depression.” Video creators were predominantly experts on TikTok (72.63%), and general users on Bilibili (56.99%). Video quality, assessed using JAMA, GQS, and mDISCERN, varied across platforms. There were statistically significant differences in the three quality scores among different types of creators on TikTok and Bilibili (*P* < 0.005). No significant differences were observed in views, likes, comments, and collections data across different video publishers on TikTok and Bilibili.

**Conclusion:**

Videos on social media platforms can help the public gain knowledge about adolescent depression. However, the quality of video from all platforms requires improvement. Strengthening collaboration among content creators, mental health experts, and platform administrators may enhance video quality and ensure more accurate and effective dissemination of information.

## 1 Introduction

Depression is a complex group of mood disorders characterized by significant and persistent emotional disturbances, often accompanied by varying degrees of cognitive and behavioral dysfunction (1, 2). This is a prevalent yet serious mood disorder (3). Depression currently ranks as the leading cause of mental health-related disability and a major contributor to the disease burden worldwide (4–6).

The adolescent stage (aged 10–19) is a sensitive developmental phase that shapes long-term mental health trajectories and represents a high-risk period for the emergence of depressive and other mood disorders (7). Studies from various regions worldwide have shown an increasing prevalence of depression among adolescents (8). Findings from The 2019 Global Burden of Disease study found that the number of adolescents with depression was estimated at 38 million in 1990, increased by 46 million in 2019, and rose by 21.67% from 1990 to 2019 (9). A study screening 546 adolescent students for depression found that one-third of them exhibited depressive symptoms (10). Symptoms such as insomnia, changes in appetite, psychomotor retardation, and other depressive symptoms can significantly impair the educational, occupational, and social functioning of adolescents (11, 12). If left untreated, depression may lead to suicidal ideation (13). Similar to most psychological disorders, early diagnosis and timely treatment are crucial for improving the prognosis of adolescent depression due to its high prevalence, persistence, potential occurrence during a crucial period of brain development, and possible long-term consequences as individuals transition into adulthood (14).

However, symptoms of depression in adolescents are often under recognized and inadequately managed (10), with only 50% of adolescents receiving a diagnosis. The general populace, including teenagers and their parents, often fail to recognize adolescent depression, tending to ignore symptoms or seek help from informal sources (15), which impedes early detection and timely intervention. Studies have demonstrated that enhancing parental awareness of depression can mitigate its negative impact on adolescents (16). Therefore, raising public awareness is a critical social issue that requires urgent attention.

In the digital era, the Internet has become an indispensable component of daily life (17), particularly among adolescents. Global representative samples have demonstrated the widespread use of the Internet to access health information (18). A significant amount of health-related information is disseminated through online platforms. The growing popularity of video-sharing platforms has made interesting and intuitive videos increasingly popular (19), TikTok, launched in 2016, and Bilibili, established in 2009, are the two most popular video platforms in China, attracting millions of active users daily (20–22). Users can create, share, comment on, repost, and engage in various other interactions with videos, they produce on TikTok and Bilibili, exerting substantial influence—particularly in how adolescents access and engage with information related to mental health, daily life, and social trends (23).

For a long time, academic research has primarily focused on uncovering the epidemiological status of adolescent depression (24), its multidimensional influencing factors (particularly social factors) (24, 25), as well as traditional and emerging intervention methods (26, 27). As video platforms such as TikTok and Bilibili have become important channels for adolescents to access mental health information, the academic community has begun to pay attention to the content characteristics and information quality of “adolescent mental health-related videos.” Preliminary studies have shown that while this information ecosystem is rich in content, it carries significant shortcomings in content quality (28, 29). Liu et al. (30) analyzed the quality and reliability of content related to body image dissatisfaction, which exerts a significant impact on adolescents. The results indicated that videos about body image dissatisfaction on YouTube are notably superior to those on TikTok and Bilibili. Additionally, other researchers have found through surveys and analyses that the quality of adolescent sex education-related videos on Chinese video platforms ranges from moderate to poor, and their reliability is questionable (31). Studies investigating short videos on other health-related topics have similarly reported comparable findings (28, 32). For example, researchers observed that the informational content and quality of videos addressing gastroesophageal reflux disease remain in need of improvement (33). To date, no researchers have conducted professional inspection and evaluation of depression-related videos targeting adolescents; some of these videos may even disseminate misleading information (34). This not only fails to contribute to adolescents’ mental health but may also delay their help-seeking behavior, exacerbate mental health stigma, or lead to erroneous behaviors. Therefore, conducting quality evaluation directly on videos focusing on the core topic of “adolescent depression” has become an urgent research task in the field of digital mental health.

In response to this need, this study utilized assessment tools, including the Journal of the American Medical Association (JAMA), Global Quality Score (GQS), and the Modified DISCERN (mDISCERN), to evaluate the top 100 videos on adolescent depression from TikTok and Bilibili. A comparative analysis was conducted to assess the quality of these videos on adolescent depression, aiming to provide the public, especially adolescents and their parents, with reliable and comprehensive resources to facilitate early detection and treatment of adolescent depression.

## 2 Methods

### 2.1 Ethics approval and information consent

Since the data from TikTok and Bilibili were publicly available, and no clinical data, human specimens, or laboratory animals were involved in this study, the ethics committee approval and informed consent were not required.

### 2.2 Video collection

The primary goal of this study was to gather video data on the topic of “adolescent depression” from Bilibili^1^ and TikTok^2^. To ensure the comprehensiveness and representativeness of the data, the Chinese term for “adolescent depression” was used as the search keyword on October 15, 2024. Data collection was carried out separately on both platforms. To minimize bias from personalized recommendations, all searches were conducted under newly created accounts, and searches were performed in an incognito mode, ensuring that no historical data or personalized algorithms influenced the results. Initially, videos ranked in the top 100 on each platform were collected based on the default sorting order. This strategy simulates the typical browsing habits of most viewers and has been shown to be feasible in prior studies (35, 36). Additionally, videos that were repetitive, irrelevant, non-Chinese, or audio-only were excluded, the remaining videos were evaluated by two authors.

### 2.3 Video characteristics

The following data of video characteristics were extracted: (1) title and Uniform Resource Locator (URL), (2) identities of video creators, (3) upload time, (4) video duration, (5) engagement data (including total play count (only Bilibili), likes, shares, comments, and collections), (6) video content. The identities of video creators were categorized as general user, expert, professional institution, news media, and health communication platform. Experts included medical professionals, specialists, researchers, and healthcare workers actively engaged in adolescent depression research and medical practices. Professional institutions cover hospitals and government healthcare organizations. General users were defined as individuals lacking relevant medical or research backgrounds. News media refer to officially qualified news organizations (e.g., People’s Daily, CCTV News, etc.). Health communication platforms are profit-oriented institutions centered on the health field (such as health management companies, pharmaceutical enterprises, commercial health apps, etc.), and their published content mainly involves the promotion of health products, the attracting traffic of paid services, and so on.

### 2.4 Video quality

This study employs three standardized scales to evaluate the informational content and quality of videos related to adolescent depression on TikTok and Bilibili. Firstly, the JAMA is used to assess the reliability of the videos (37). This scale consists of four core criteria: authorship, attribution, disclosure, and currency (38), as detailed in Table 1. Each positive response receives a score of 1, while each negative response receives a score of 0. The highest possible score is 4, indicating the highest level of accuracy and reliability (35). Secondly, the GQS is adopted to assess the quality of the educational content (39). The five questions on this scale are listed in Table 2, with each question scored on a range from 1 to 5, where a higher score represents higher quality (40). Lastly, the mDISCERN tool is utilized to analyze both the reliability and quality of the videos. This tool is based on five binary (yes/no) questions, as shown in Table 3. One point is awarded for each “Yes” response and zero points for each “No” response. A maximum score of 5 across all five questions indicates high reliability (28). The average score assigned by two authors to each video was considered as the final score for the video. In cases of disagreement, the final decision was made through thorough discussion with the third author. Prior to formal rating, all raters received systematic training on the operational definitions of each item in JAMA, GQS, and mDISCERN to standardize their understanding.

**Table 1:** JAMA criteria.

Criterion	Description
Authorship	Authors and contributors, their affiliations, and relevant credentials should be provided
Attribution	References and sources for all content should be listed clearly, and all relevant copyright information noted
Disclosure	Ownership,” sponsorship, advertising, underwriting, commercial funding arrangements or support, or potential conflicts of interest should be prominently and fully disclosed
Currency	Dates that content was posted and updated should be indicated

**Table 2:** GQS criteria.

Item	Description
1	Poor quality and flow, most information missing; technique misleading; unlikely to be useful for patient education
2	Generally sparse quality and fow, some information provided but many important topics missing; technique poor; of very limited use to patients
3	Moderate quality and suboptimal fow, some important information provided adequately but others poorly discussed; technique basically adequate; somewhat useful for patients
4	Good quality and generally good fow, majority of information provided but some topics not covered; technique almost adequate; useful for patients
5	Excellent quality and fow, full information provided; technique adequate; highly useful for patients

**Table 3:** mDISCERN criteria.

Item	Description
1	Are the aims clear and achieved?
2	Are reliable sources of information used? (i.e., publication cited; provided by certified orthopedists or neurosurgeons)
3	Is the information presented balanced and unbiased?
4	Are additional sources of information listed for patient reference?
5	Are areas of uncertainty mentioned?

### 2.5 Statistical analysis

IBM SPSS Statistics 25.0 was used to analyze the data. Continuous variables are presented as mean ± standard deviation or median and interquartile range, depending on the distribution. Categorical variables are expressed as frequencies and percentages. The independent *t*-test was employed to compare quantitative variables with a normal distribution. For comparing multiple sets of data with quantitative variables that follows a normal distribution and has homogeneous variances, one-way ANOVA was employed; otherwise, the Kruskal–Wallis test was used. All statistical tests were two-sided, and statistical significance was considered at a *p*-value of <0.05.

## 3 Results

### 3.1 Overview of the video screening process

**Figure 1:**
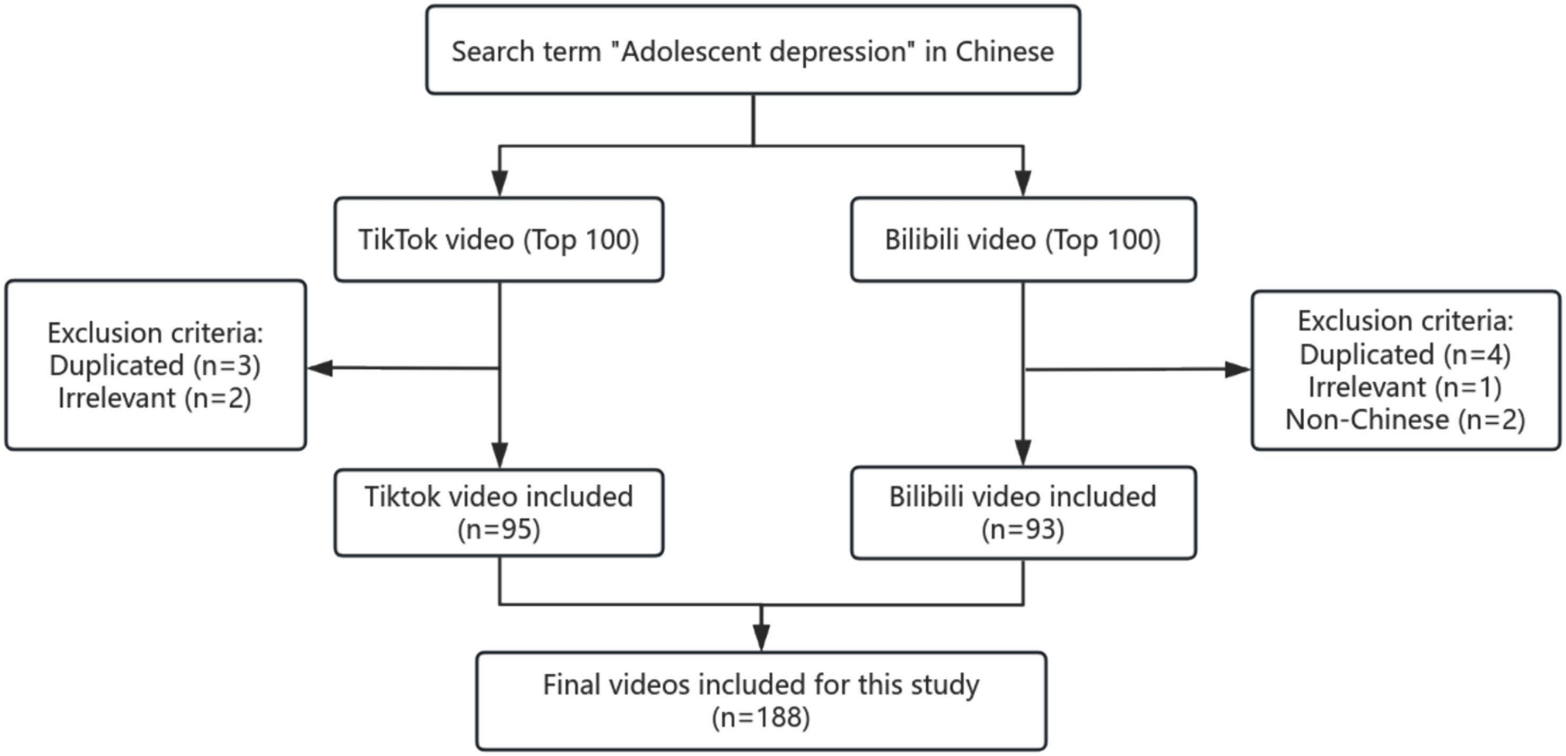
Search strategy for videos on adolescent depression.

A total of 200 videos on adolescent depression were retrieved for evaluation from the online platforms TikTok and Bilibili. For TikTok, 3 duplicate and 2 irrelevant videos were removed; for Bilibili, 4 duplicate videos, 1 irrelevant, and 2 videos in non-Chinese languages were excluded. Consequently, a refined collection of 95 relevant videos from TikTok and 93 suitable videos from Bilibili were included in the analysis. Ultimately, 188 eligible videos from both TikTok and Bilibili were evaluated (Figure 1).

### 3.2 Upload time of the videos on TikTok/Bilibili

**Figure 2:**
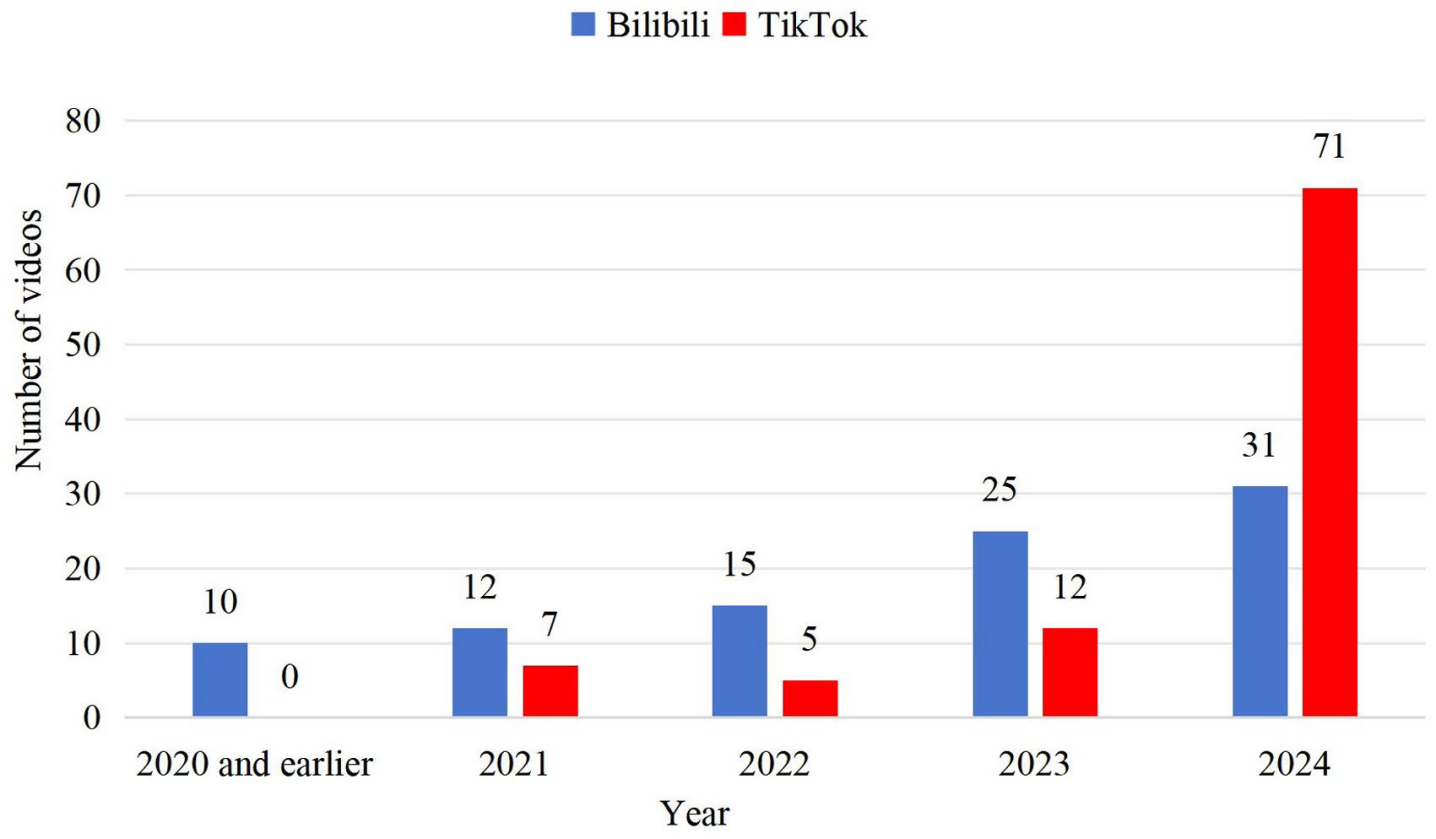
A line chart shows 188 adolescent depression related videos on TikTok/Bilibili.

The 95 videos included in the TikTok platform were first released on October 21, 2021, and the most recent video was uploaded on October 30, 2024. Among the 93 videos from the Bilibili platform, the earliest released on September 18, 2016, and the latest was uploaded on October 10, 2024. On Bilibili, the distribution of videos by year was as follows: 10 videos from 2020 and earlier (10.75%), 12 videos from 2021 (12.90%), 15 videos from 2022 (16.23%), and 25 videos from 2023 (26.88%), 31 videos from 2024 (33.33%), were retrieved on Bilibili (Figure 2). In contrast, on TikTok, 7 videos from 2021 (7.37%), 5 from 2022 (5.26%), 12 from 2023 (12.63%), and 71 from 2024 (74.74%) (Figure 2).

### 3.3 Video duration and interaction

The video duration on the two platforms was evaluated. As a short-video platform, TikTok had significantly shorter video duration than Bilibili (*p* < 0.001). However, the TikTok videos outperformed the Bilibili videos in terms of comments and shares, with significantly higher engagement (*p* < 0.001) (Table 4). In summary, Bilibili tends to feature longer videos released, while TikTok offers more interactive content (Table 4).

**Table 4:** Detailed characteristics of adolescent depression videos on TikTok/Bilibili.

Variable	TikTok (*n* = 95)	Bilibili (*N* = 93)	*p*-value
Duration (median) (IQR)	124 (10, 6,018)	507 (69, 4,615)	<0.001
Views (median) (IQR)	N/A	11,000 (18,10,243,000)	N/A
Likes (median) (IQR)	794 (3,208,000)	325 (0,86,200)	0.060
Comments (median) (IQR)	58 (0,22,000)	52 (0,36,854)	<0.001
Shares (median) (IQR)	232 (0,61,000)	120 (0,541,000)	<0.001
Collections (median) (IQR)	219 (0,66,000)	312 (0,393,000)	0.199
JAMA score (mean ± SD)	2.84 ± 0.66	2.12 ± 0.90	0.003
GQS score (mean ± SD)	2.71 ± 0.74	3.04 ± 0.98	0.008
mDISCERN score (mean ± SD)	1.98 ± 0.99	1.55 ± 0.95	<0.001

N/A, not applicable.

### 3.4 Analysis of video content

In this study, video content was categorized into 10 types, including topics such as characteristics of adolescents, definition of depression, and incidence rate (Table 5). The most prevalent content category across both platforms was “symptoms of adolescents depression,” with TikTok covering this topic in 37.89% of videos, and Bilibili in 53.76%. The second most popular content on Bilibili focused on “etiology and pathogenesis” (46.24%) and “prevention and intervention” (46.24%). In contrast, TikTok videos were more likely to cover “parental practices” (36.84%), “patient narratives” (36.84%), and “case analyses” (21.05%) (Table 5).

**Table 5:** Descriptions of video content.

Content	TikTok (*n*, %)	Bilibili (*n*, %)
Characteristics of adolescents	2 (2.11)	26 (27.96)
Definition of depression	2 (2.11)	16 (17.20)
Incidence rate	18 (18.95)	28 (30.11)_
Symptoms of adolescents depression	36 (37.89)	50 (53.76)
Etiology and pathogenesis	19 (20.00)	43 (46.24)
Diagnosis and treatment	13 (13.68)	30 (32.26)
Prevention and intervention	15 (15.79)	43 (46.24)
Parental practices	35 (36.84)	16 (17.20)
Patient narratives	20 (21.05)	13 (13.98)
Self-regulation	9 (9.47)	17 (18.28)
Case analyses	20 (21.05)	26 (27.96)

### 3.5 Identities of video creators

**Figure 3:**
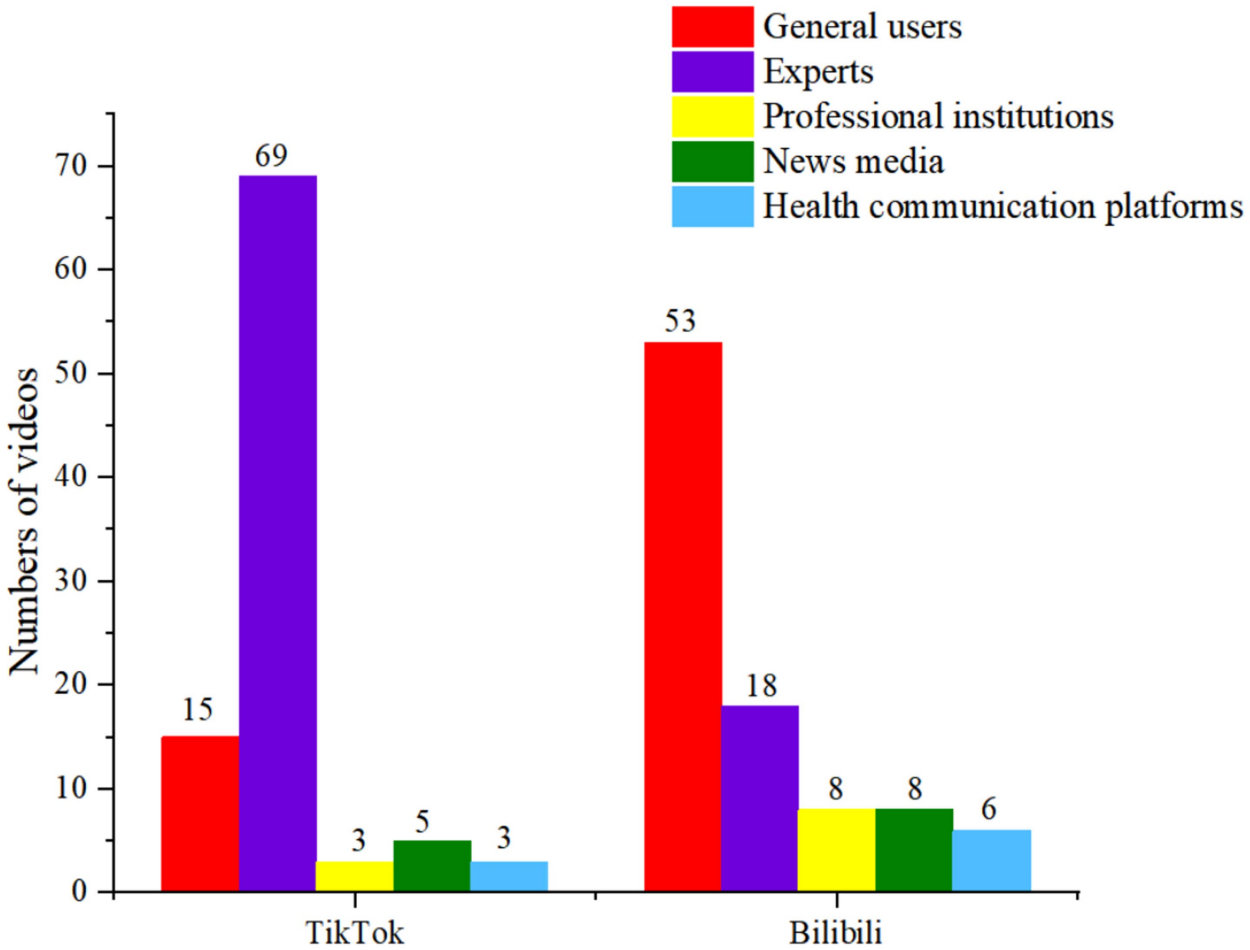
Numbers of video creators about adolescent depression on TikTok/Bilibili.

Due to the largely unrestricted nature of video uploads on both TikTok and Bilibili, a wide variety of users contribute content. The results showed that a significant majority of adolescent depression related videos on TikTok were uploaded by experts (69, 72.63%). The remaining contributors were general users (15, 15.79%), news media (5, 5.26%), professional institutions (3, 3.16%), and health communication platforms (3, 3.16%). On the other hand, Bilibili had a higher proportion of videos from general users (53, 56.99%), followed by professionals (18, 19.35%), professional institutions (8, 8.60%), news media (8, 8.60%), and health communication platforms (6, 6.45%). In general, TikTok had more professional contributions in terms of adolescent depression-related videos, while Bilibili featured more content from general users (Figure 3).

### 3.6 Assessment of video quality

**Figure 4:**
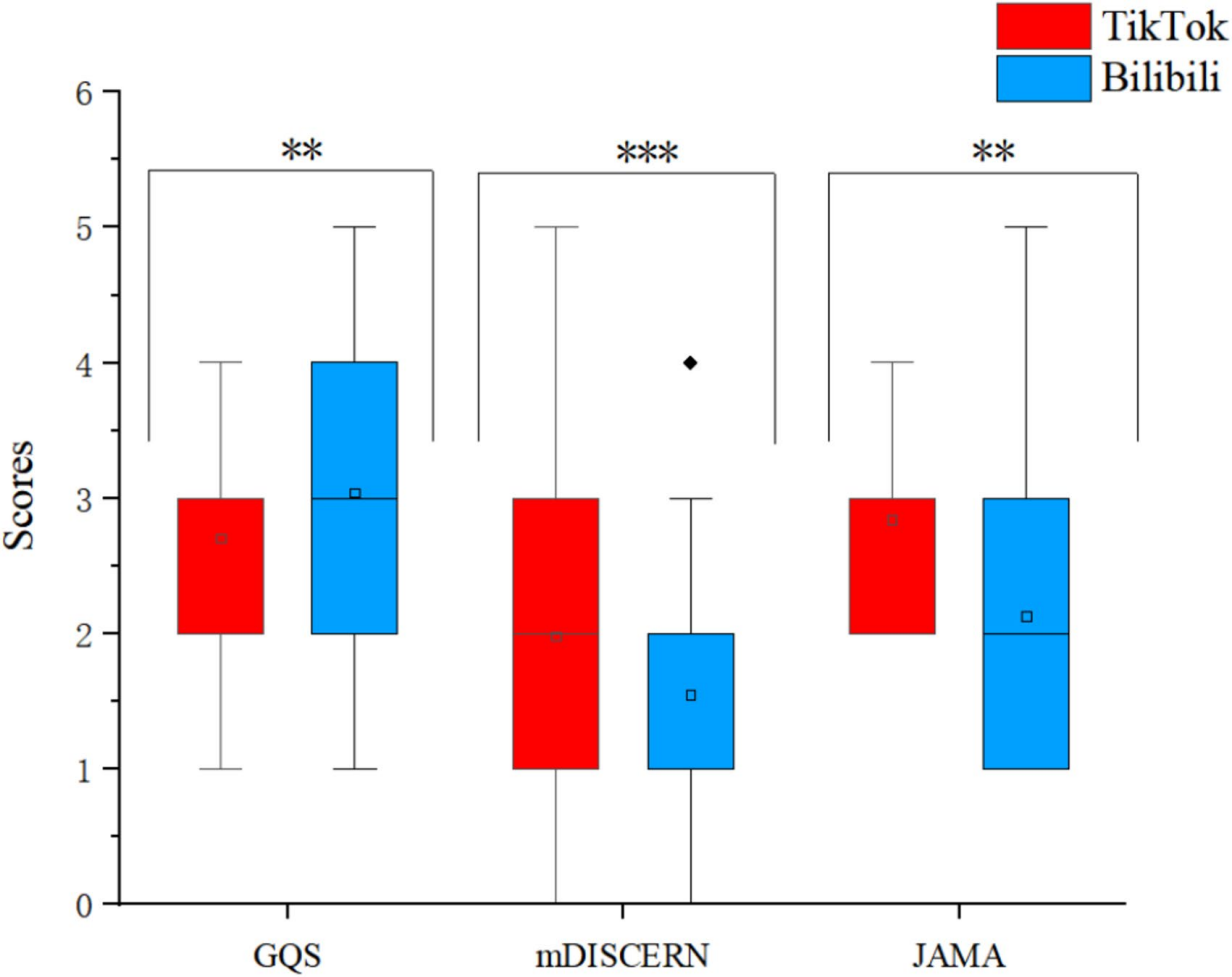
The box graph shows a comparison of GQS, MDISCERN, and JAMA scores on TikTok/Bilibili.

According to the rating criteria of GQS and JAMA, a majority of TikTok videos fall into the “fair” category, accounting for 55.79 and 58.95%, respectively. Additionally, the majority of Bilibili videos, which have the highest proportion, also belong to this category (37.63 and 45.16% respectively). As for the mDISCERN, 47.37% of TikTok videos and 38.71% of Bilibili were rated as “Slightly reliable” (Table 6). The JAMA score for TikTok videos was 2.84 points, compared to 2.12 points for Bilibili videos. Using the mDISCERN score, which assesses the usability and reliability of the videos, TikTok scored 1.98 points, while Bilibili scored 1.55 points. These scores suggest that TikTok’s videos were overall of higher quality than those on Bilibili (*p* < 0.05). However, when assessed using the GQS, Bilibili’s video (3.04) quality surpasses that of TikTok (2.71) (*p* < 0.05), the score distributions are compared in Figure 4.

**Table 6:** The results of the JAMA, GQS and mDISCERN of videos on TikTok/Bilibili.

Scale, score	TikTok (*n* = 95), *n* (%)	Bilibili (*n* = 93), *n* (%)
JAMA		
0 (very poor)	5 (5.26)	0 (0)
1 (poor)	17 (17.89)	29 (31.83)
2 (fair)	56 (58.95)	42 (45.16)
3 (good)	17 (17.89)	21 (22.58)
4 (excellent)	0 (0)	1 (1.08)
GQS		
1 (very poor)	6 (6.32)	5 (5.38)
2 (poor)	26 (27.37)	22 (23.66)
3 (fair)	53 (55.79)	35 (37.63)
4 (good)	10 (10.53)	26 (27.96)
5 (excellent)	0 (0)	5 (5.38)
mDISCERN		
0 (Unreliable)	9 (9.47)	10 (10.75)
1 (Less reliable)	16 (16.84)	35 (37.63)
2 (Slightly reliable)	44 (46.32)	36 (38.71)
3 (Relatively reliable)	21 (22.11)	11 (11.83)
4 (Highly reliable)	4 (4.21)	1 (1.8)
5 (Extremely Reliable)	1 (1.05)	0 (0)

### 3.7 Relationship between creator and quality score

**Figure 5:**
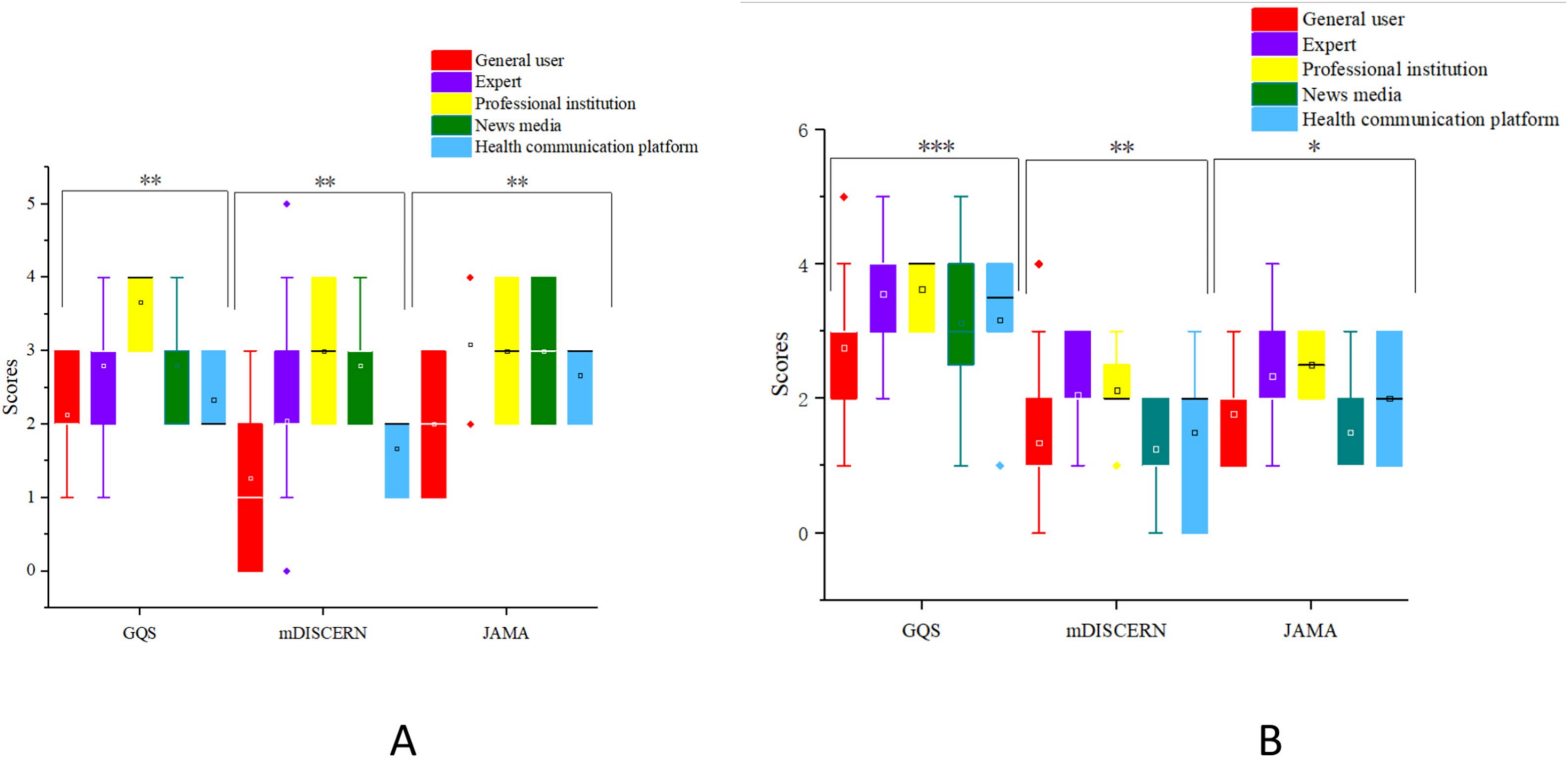
The box graph shows a comparison of creators on GQS, MDISCERN, and JAMA scores [**(A)** is comparison on TikTok, **(B)** is comparison on Bilibili].

On TikTok, professional institutions received the highest score for both the GQS and mDISCERN, with scores of 3.67 and 3.00, respectively (*p* < 0.05). When using the JAMA, expert receive the highest score (3.09, *p* < 0.05). On Bilibili, there are also significant differences in three quality scores among different types of uploads, with the professional institutions scoring the highest score (*p* < 0.05) (Figure 5).

### 3.8 Relationship between creator and engagement data

A further comparison of engagement data, including the number of views, likes, comments, shares, and collections, showed no significant differences between different video creators on either TikTok or Bilibili (Tables 7, 8).

**Table 7:** Comparison of creators according to engagement data on TikTok.

Video features	General user	Expert	Professional institution	News media	Health communication platform	*p*-value
Duration (median) (range)	167 (28,764)	118 (13,6,018)	65 (63,376)	130 (25,1800)	179 (88,554)	0.187
Likes (median) (range)	580 (30,208,000)	537 (3,188,000)	499 (282,7,965)	2,134 (580,188,000)	2,330 (26,27,000)	0.368
Comments (median) (range)	137 (1,22,000)	50 (0,12,000)	24 (20,1,027)	156 (46,12,000)	182 (10,681)	0.222
Shares (median) (range)	123 (9,50,000)	272 (0,56,000)	395 (74,10,000)	278 (64,56,000)	992 (12,7,137)	0.251
Collections (median) (range)	169 (7,61,000)	242 (0,38,000)	173 (110,8,146)	606.5 (169,38,000)	1,270 (6,6,749)	0.284

**Table 8:** Comparison of creators according to engagement data on Bilibili.

Video features	General user	Expert	Professional institution	News media	Health communication platform	*p*-value
Duration (median) (range)	500 (96,4,615)	448.5 (96,1708)	696.5 (69,2,151)	536.5 (164,1,437)	824.5 (92,1810)	0.995
Views (median) (range)	18,000 (18,4,840,000)	5132.5 (97,102,000)	55,500 (28,380,000)	64,500 (39,10,243,000)	17999.5 (1,420,1,001,000)	0.622
Likes (median) (range)	573 (0,210,000)	126 (1,6,222)	1874.5 (1,14,000)	1,335 (1,862,000)	221 (15,73,000)	0.451
Comments (median) (range)	444 (0,106,000)	108 (0,4,575)	1616.5 (1,12,000)	2213.5 (1,393,000)	513 (30,25,000)	0.290
Shares (median) (range)	126 (0,14,997)	16.5 (0,1,170)	149.5 (0,1,273)	377.5 (0,26,854)	26.5 (1,4,297)	0.661
Collections (median) (range)	166 (0,53,000)	52 (0,2,374)	301 (0,7,520)	1918 (0,241,000)	156.5 (27,12,000)	0.858

## 4 Discussion

In the context of the widespread popularity of the Internet and new media, video platforms have become important channels for accessing health information (16, 18). With the increasing demand for health knowledge among the public, these platforms have transformed the role of patients from passive recipients to active explorers (18). However, existing studies have shown that there are still obvious deficiencies in the overall quality of video content on these platforms (28, 41). In China’s short video platforms, TikTok and Bilibili rank first with their large user bases (19, 21). This study provides valuable insights for improving the public’s understanding of adolescent depression by evaluating the quality of videos related to adolescent depression on two video platforms, TikTok and Bilibili, and calls on medical expert to take responsibility for enhancing the quality of health content (39).

In this study, a total of 188 videos were analyzed. The number of videos from 2024 on TikTok is significantly higher than on Bilibili, indicating that TikTok has a stronger tendency to feature videos from the most recent year. This difference can be attributed to the algorithmic variations between the two platforms, where the publication date is given greater priority during the search process on TikTok.

The video duration on TikTok is generally shorter than that on Bilibili, which is also a prominent feature of videos on the TikTok. This time constraint, to a certain extent, leads to simpler and less in-depth content, rather than specialized and systematic medical knowledge. This makes TikTok videos more intuitive, emotional, easier to watch and share, and more likely to evoke resonance and discussions. This, to some extent, contributes to TikTok’s better engagement data compared to Bilibili. On the other hand, TikTok’s content recommendation mechanism employs a decentralized algorithm that effectively pushes content tailored to users’ interests. When users demonstrate interest in a particular type of video (e.g., adolescent depression), TikTok continuously recommends related content, thereby enhancing interaction on the platform.

This study categorizes the content attributes of videos into 10 categories based on their content, which indicates that the content of videos related to adolescent depression from platforms like TikTok and Bilibili is very diverse and can basically meet the public’s demand for knowledge about the disease. Among them, videos related to depressive symptoms in adolescents are the most numerous. Video creators tended to provide more knowledge about how to identify symptoms of adolescent depression. Consistent with our research, symptoms have also been the most prevalent theme in previous studies on other diseases such as breast cancer and gastric cancer (42–44). This may be effective for the early identification and treatment of depression among adolescents, as studies have shown that the identification and treatment rates for depression are very low (45). On Bilibili, there is a significant amount of content related to the “etiology and pathogenesis” and “prevention and intervention” of adolescent depression. In contrast, video creators on TikTok focus more on how parents should respond to adolescent depression, indicating that the TikTok short video platform is closer to public life (46). This suggests a potential strategy for video creators to increase audience interaction.

The three most common scoring systems were utilized to assess the videos on TikTok and Bilibili. Overall, most videos were rated as medium or even low quality, which aligns with existing research (34, 41), indicating a subpar performance across the board (34). To address this, TikTok and Bilibili should establish professional review teams to strictly scrutinize video content related to adolescent depression, ensuring its accuracy and scientific rigor. Experts in psychology, psychiatry, and related fields should be invited to evaluate video content and provide authoritative guidance. At the same time, collaboration among content creators, mental health experts, and platform administrators should be enhanced. Video platforms also could establish dedicated “health” modules that only allow the publication of videos subjected to verification or professional review. Such modules can effectively alleviate the growing concerns over misinformation and its potential harm to public health (28). In comparison between the two platforms, the quality of videos on TikTok generally appears superior to that on Bilibili, this discrepancy may be attributed to the higher proportion of professional video creators on TikTok. These professionals tend to produce more authoritative, and scientifically grounded content, consistenting with previous research findings (47, 48), while Bilibili’s user base primarily consists of ordinary users. However, the GQS scores indicate that TikTok’s quality is inferior to that of Bilibili, possibly due to the shorter of TikTok videos, the GQS focuses on evaluating the comprehensiveness of the video content, which may not present content comprehensively and thus receive lower scores.

To our surprise, there were no significant differences in the level of interaction among videos from different creators. It seems that the public may not be discerning videos based on the creator’s expertise. However, given the high professional standards required for medical science popularization, some researchers argue that short video platforms should set specific thresholds for video creators (19). We concur with this recommendation, as it could help improve the quality of information. Official platforms could implement professional certifications to verify the identities of video creators, thereby reducing the spread of false or unscientific content.

This study has several limitations that must be acknowledged. Firstly, in consideration of the public’s language proficiency, we excluded videos that are not in Chinese. Second, a notable limitation of this study is its cross-sectional design, which fails to track the temporal dynamics of these metrics. Future longitudinal studies could be conducted, with a primary focus on examining the evolutionary trajectories of video characteristics and quality metrics. Additionally, despite rigorous measures to standardize rating and verify inter-rater reliability, the subjective nature of tools like GQS and mDISCERN means residual bias from individual rater interpretation cannot be fully eliminated, which may have marginally influenced final quality scores. Lastly, video creators have the right to modify or delete their videos, which may introduce bias into the search results.

## 5 Conclusion

In this study, we collected and assessed the information quality of 188 videos related to adolescent depression on two major social media platforms in China (TikTok and Bilibili). The videos related to adolescent depression on these platforms cover a wide range of content, such as symptoms, prevention, treatment, parental actions, and play a crucial role in popularizing knowledge about adolescent depression. However, the quality and reliability of these videos are considerably inadequate. Consequently, there is a pressing need to enhance the regulation and quality control of video platforms to guarantee the public’s access to accurate, reliable, and scientifically supported mental health content. Furthermore, research should explore ways to foster collaboration between content creators, mental health experts, and platform administrators to improve the quality of health-related videos, thereby supporting the early detection and timely treatment of adolescent depression.

## Data Availability

The raw data supporting the conclusions of this article will be made available by the authors, without undue reservation.

## Footnotes

1
https://www.bilibili.com

2
https://www.douyin.com

## References

[1] Su WJ Liu H Zhou XY Huang XP. Depression and non-suicidal self-injury: the mediating roles of childhood trauma and impulsivity. Front Psych. (2025) 16:1580235. doi: 10.3389/fpsyt.2025.1580235, PMID: 40621556 PMC12226557

[2] Malhi GS Mann JJ. Depression. Lancet. (2018) 392:2299–312. doi: 10.1016/S0140-6736(18)31948-2, PMID: 30396512

[3] Li M Gao WL Zhang YQ Luo QX Xiang YY Bao K . Secular trends in the incidence of major depressive disorder and dysthymia in China from 1990 to 2019. BMC Public Health. (2023) 23:2162. doi: 10.1186/s12889-023-17025-4, PMID: 37926849 PMC10626640

[4] Korczak DJ Westwell-Roper C Sassi R. Diagnosis and management of depression in adolescents. CMAJ. (2023) 195:E739–e746. doi: 10.1503/cmaj.220966, PMID: 37247881 PMC10228578

[5] Krause KR Chung S Adewuya AO Albano AM Babins-Wagner R Birkinshaw L . International consensus on a standard set of outcome measures for child and youth anxiety, depression, obsessive-compulsive disorder, and post-traumatic stress disorder. Lancet Psychiatry. (2021) 8:76–86. doi: 10.1016/S2215-0366(20)30356-4, PMID: 33341172

[6] Lu BQ Lin LX Su XJ. Global burden of depression or depressive symptoms in children and adolescents: a systematic review and meta-analysis. J Affect Disord. (2024) 354:553–62. doi: 10.1016/j.jad.2024.03.074, PMID: 38490591

[7] Huang HC Zhang YN Wu XY Jiang Y Cai H Deng YQ . A cross-sectional study: family communication, anxiety, and depression in adolescents: the mediating role of family violence and problematic internet use. BMC Public Health. (2023) 23:1747. doi: 10.1186/s12889-023-16637-0 37679728 PMC10485963

[8] Liu G Liu W Wen J Liu Y Jin H Li Z . Evaluating the tree drawing test depression assessment scale for adolescent depression screening. Sci Rep. (2025) 15:20984. doi: 10.1038/s41598-025-99254-8, PMID: 40594844 PMC12217084

[9] Yang CH Lv JJ Kong XM Chu F Li ZB Lu W . Global, regional and national burdens of depression in adolescents and young adults aged 10-24 years, from 1990 to 2019: findings from the 2019 global burden of disease study. Br J Psychiatry. (2024) 225:311–20. doi: 10.1192/bjp.2024.69, PMID: 38660761

[10] Girma S Tsehay M Mamaru A Abera M. Depression and its determinants among adolescents in Jimma town, Southwest Ethiopia. PLoS One. (2021) 16:e0250927. doi: 10.1371/journal.pone.0250927, PMID: 33939748 PMC8092653

[11] Shorey S Ng ED Wong CH. Global prevalence of depression and elevated depressive symptoms among adolescents: a systematic review and meta-analysis. Br J Clin Psychol. (2022) 61:287–305. doi: 10.1111/bjc.12333, PMID: 34569066

[12] The National Institute of Mental Health (NIMH) (2018) Depression. Available online at: https://www.nimh.nih.gov/health/topics/depression/index.shtml (accessed April 10, 2024)

[13] Gao L Xie YC Jia CH Wang W. Prevalence of depression among Chinese university students: a systematic review and meta-analysis. Sci Rep. (2020) 10:15897. doi: 10.1038/s41598-020-72998-1, PMID: 32985593 PMC7522998

[14] Beck A LeBlanc JC Morissette K Hamel C Skidmore B Colquhoun H . Screening for depression in children and adolescents: a protocol for a systematic review update. Syst Rev. (2021) 10:24. doi: 10.1186/s13643-020-01568-3, PMID: 33436094 PMC7802305

[15] Singh S Zaki RA Farid ND. A systematic review of depression literacy: knowledge, help-seeking and stigmatising attitudes among adolescents. J Adolesc. (2019) 74:154–72. doi: 10.1016/j.adolescence.2019.06.004, PMID: 31216495

[16] Johnco C Rapee RM. Depression literacy and stigma influence how parents perceive and respond to adolescent depressive symptoms. J Affect Disord. (2018) 241:599–607. doi: 10.1016/j.jad.2018.08.062, PMID: 30172212

[17] Marzouk S Velasquez DE Joseph N Martin A. Broadband for better health-ensuring internet access for all. BMJ. (2023) 382:1673. doi: 10.1136/bmj.p1673, PMID: 37474205

[18] Lahti H Lyyra N Hietajärvi L Villberg JJ Paakkari L. Profiles of internet use and health in adolescence: a person-oriented approach. Int J Environ Res Public Health. (2021) 18:18. doi: 10.3390/ijerph18136972, PMID: 34209886 PMC8296941

[19] Zheng SS Tong XY Wan DL Hu C Hu Q Ke QH. Quality and reliability of liver cancer-related short Chinese videos on TikTok and Bilibili: cross-sectional content analysis study. J Med Internet Res. (2023) 25:e47210. doi: 10.2196/47210, PMID: 37405825 PMC10357314

[20] Richards A. Scientific communication on TikTok. Cell. (2022) 185:3066–9. doi: 10.1016/j.cell.2022.07.015 35985281

[21] Gao YC Liu FM Gao L. Echo chamber effects on short video platforms. Sci Rep. (2023) 13:6282. doi: 10.1038/s41598-023-33370-1, PMID: 37072484 PMC10111082

[22] Du RC Zhang Y Wang MH Lu NH Hu Y. TikTok and Bilibili as sources of information on *Helicobacter pylori* in China: a content and quality analysis. Helicobacter. (2023) 28:e13007. doi: 10.1111/hel.13007, PMID: 37452727

[23] Zeng FY Zhang WL Wang MH Zhang HJ Zhu XY Hu H. Douyin and Bilibili as sources of information on lung cancer in China through assessment and analysis of the content and quality. Sci Rep. (2024) 14:20604. doi: 10.1038/s41598-024-70640-y, PMID: 39232044 PMC11375008

[24] Wang Y Zhang X Li Y Qin H Li X. Gender differences in the prevalence, correlated factors and comorbidity of depression in adolescents: a cross-sectional study in Shanghai, China. Front Public Health. (2024) 12:1436413. doi: 10.3389/fpubh.2024.1436413 39712306 PMC11659128

[25] Xian X Wang R Wu Y Shi Q Zeng L Niu T. Associations of loneliness and sleep chronotype with depressive symptoms: a structural equation modeling approach. Chronobiol Int. (2025) 8:1–10. doi: 10.1080/07420528.2025.2571193, PMID: 41058432

[26] Luo S Mei Z Fang G Mu G Zhang X Luo S. Effects of mind-body therapies on depression among adolescents: a systematic review and network meta-analysis. Front Public Health. (2024) 12:1431062. doi: 10.3389/fpubh.2024.1431062, PMID: 39050611 PMC11266190

[27] Rose-Clarke K Sonmez CC Shrestha S Lamichhane B Pradhan I Pandey P . School-based group interpersonal therapy for adolescents with depression in Nepal: protocol for a phase III realist cluster-randomised controlled trial. BMC Psychiatry. (2025) 25:863. doi: 10.1186/s12888-025-07302-4, PMID: 40999412 PMC12465168

[28] Zhou X Ma G Su X Li X Wang W Xia L . The reliability and quality of short videos as health information of guidance for lymphedema: a cross-sectional study. Front Public Health. (2025) 12:1472583. doi: 10.3389/fpubh.2024.1472583, PMID: 39830188 PMC11739071

[29] Luo C Qin X Xie X Gao J Wu Y Liang W . Cross-platform analysis of atrial fibrillation scientific videos: using composite index and a basic assessment scale. Front Public Health. (2025) 13:1507776. doi: 10.3389/fpubh.2025.1507776, PMID: 40352855 PMC12061937

[30] Liu L Yang J Tan F Luo H Chen Y Zhao X. Web-based video platforms as sources of information on body image dissatisfaction in adolescents: content and quality analysis of a cross-sectional study. JMIR Form Res. (2025) 9:e71652. doi: 10.2196/71652, PMID: 40955848 PMC12439227

[31] Wang L Shu X Huang J Yan W Zhao D. Quality and reliability of adolescent sexuality education on Chinese video platforms: sentiment-topic analysis and cross-sectional study. JMIR Form Res. (2025) 9:e77100. doi: 10.2196/77100, PMID: 40910701 PMC12449667

[32] Pan C Cheng L Zhang B Hu X Wang W Jiang G. Professional content analysis and quality assessment of cardiopulmonary resuscitation educational videos on social media platforms: a comparative study of YouTube, BiliBili, and TikTok. Front Public Health. (2025) 15:1657233. doi: 10.3389/fpubh.2025.1657233 PMC12477029 41030356

[33] Cai Y Zeng H Yang P Xu X Lai Y Zhou Y. The status quo of short video as sources of health information on gastroesophageal reflux disease in China: a cross-sectional study. Front Public Health. (2024) 28:1400749. doi: 10.3389/fpubh.2024.1400749 PMC11165113 38864023

[34] Niu ZY Hao YJ Yang FJ Jiang QR Jiang YP Zhang SZ . Quality of pancreatic neuroendocrine tumor videos available on TikTok and Bilibili: content analysis. JMIR Form Res. (2024) 8:e60033. doi: 10.2196/60033, PMID: 39661988 PMC11655045

[35] Kunze KN Krivicich LM Verma NN Chahla JC. Quality of online video resources concerning patient education for the Meniscus: a YouTube-based quality-control study. Arthroscopy. (2020) 36:233–8. doi: 10.1016/j.arthro.2019.07.033, PMID: 31864582

[36] Wang H Yan CY Wu TK Zhang X He JB Liu ZH . YouTube online videos as a source for patient education of cervical spondylosis-a reliability and quality analysis. BMC Public Health. (2023) 23:1831. doi: 10.1186/s12889-023-16495-w, PMID: 37730621 PMC10512502

[37] He WJ Tang DN Jin Y Zhang WY Kang YY Xia Q. Quality of cerebral palsy videos on Chinese social media platforms. Sci Rep. (2025) 15:13323. doi: 10.1038/s41598-024-84845-8, PMID: 40246856 PMC12006377

[38] Ozsoy HE. Evaluation of YouTube videos about smile design using the DISCERN tool and journal of the American Medical Association benchmarks. J Prosthet Dent. (2021) 125:151–4. doi: 10.1016/j.prosdent.2019.12.016, PMID: 32085870

[39] Li ZX Lin YS Zhang KR Li R Ju M Chen YH . Hip fractures in Chinese TikTok (Douyin) short videos: an analysis of information quality, content and user comment attitudes. Front Public Health. (2025) 13:1563188. doi: 10.3389/fpubh.2025.1563188, PMID: 40342510 PMC12058786

[40] Jiang SS Zhou YL Qiu J Gou XH. Search engines and short video apps as sources of information on acute pancreatitis in China: quality assessment and content assessment. Front Public Health. (2025) 13:1578076. doi: 10.3389/fpubh.2025.1578076, PMID: 40535445 PMC12174125

[41] Zhang R Zhang ZW Jie H Guo Y Liu Y Yang Y . Analyzing dissemination, quality, and reliability of Chinese brain tumor-related short videos on TikTok and Bilibili: a cross-sectional study. Front Neurol. (2024) 15:1404038. doi: 10.3389/fneur.2024.1404038, PMID: 39494168 PMC11527622

[42] Wang MH Yao N Wang JM Chen WJ Ouyang YB Xie C. Bilibili, TikTok, and YouTube as sources of information on gastric cancer: assessment and analysis of the content and quality. BMC Public Health. (2024) 24:57. doi: 10.1186/s12889-023-17323-x, PMID: 38166928 PMC10763378

[43] Yang SJ Zhan J Xu XQ. Is TikTok a high-quality source of information on thyroid cancer? Endocrine. (2023) 81:270–6. doi: 10.1007/s12020-023-03332-8, PMID: 36840912

[44] Liu H Peng JL Li L Deng A Huang XX Yin GB . Assessment of the reliability and quality of breast cancer related videos on TikTok and Bilibili: cross-sectional study in China. Front Public Health. (2023) 11:1296386. doi: 10.3389/fpubh.2023.1296386, PMID: 38317686 PMC10839971

[45] Trivedi MH. Major depressive disorder in primary care: strategies for identification. J Clin Psychiatry. (2020) 81:81. doi: 10.4088/JCP.UT17042BR1C, PMID: 32220155

[46] Liu ZY Chen YW Lin Y Ai MM Lian DL Zhang YH . YouTube/Bilibili/TikTok videos as sources of medical information on laryngeal carcinoma: cross-sectional content analysis study. BMC Public Health. (2024) 24:1594. doi: 10.1186/s12889-024-19077-6, PMID: 38877432 PMC11177428

[47] Xia L Bao J Yao K Zhang J Yu W. Evaluation of the quality and reliability of Chinese content about orthognathic surgery on BiliBili and TikTok: a cross-sectional study. Sci Rep. (2025) 15:28967. doi: 10.1038/s41598-025-13941-0, PMID: 40775506 PMC12332024

[48] Li Q Jin L Shi K Zheng X. Short-video platforms as sources of atherosclerosis information: a cross-sectional content analysis. Medicine. (2025) 104:e45006. doi: 10.1097/MD.0000000000045006, PMID: 41054099 PMC12499849

